# Knoellia altitudinis sp. nov., Knoellia pratensis sp. nov., Knoellia terrae sp. nov. and Knoellia tibetensis sp. nov., four novel UV radiation-resistant actinobacteria isolated from Tibet Autonomous Region, China

**DOI:** 10.1099/ijsem.0.006855

**Published:** 2025-08-08

**Authors:** Jing Zhang, Tong Mou, Cong-Jian Li, Jing-Lin Bai, Li-Yan Yu, Hua-Hong Chen, Yu-Qin Zhang

**Affiliations:** 1 Institute of Medicinal Biotechnology, Chinese Academy of Medical Sciences & Peking Union Medical College, Beijing 100050, PR China; 2 College of Resources, Environmental Sciences and Chemistry, Chuxiong Normal University, Chuxiong, Yunnan, 675000, PR China

**Keywords:** genome, *Knoellia*, polyphasic taxonomy, UV radiation tolerance

## Abstract

Four Gram-stain-positive, aerobic, non-motile and non-spore-forming actinobacterial strains (CPCC 206391^T^, CPCC 206453^T^, CPCC 206435^T^ and CPCC 206450^T^) were isolated from soil samples collected from Tibet Autonomous Region, China. The 16S rRNA gene sequences of these four strains showed close relations to members of the genus *Knoellia* of the family *Intrasporangiaceae*, with similarities of 96.1–99.4% to the validly named species of *Knoellia*. In the phylogenetic trees based on 16S rRNA gene sequences and the core genome, these isolates clustered into the genus *Knoellia* clade within the lineage of the family *Intrasporangiaceae*. Genome relatedness index values between these strains and their phylogenetic neighbours, including average nucleotide identity (78.1–91.5%) and digital DNA–DNA hybridization (19.8–41.0%), were all below the species delineation thresholds. These genomic data, combined with their phenotypic characteristics, supported their classification within the genus *Knoellia*, representing four novel species. Thereby, *Knoellia altitudinis* sp. nov. (type strain CPCC 206391^T^=XZ253^T^=KCTC 59139^T^), *Knoellia pratensis* sp. nov. (type strain CPCC 206453^T^=CXZ644^T^=KCTC 59274^T^), *Knoellia terrae* sp. nov. (type strain CPCC 206435^T^=CXZ904^T^=KCTC 59271^T^) and *Knoellia tibetensis* sp. nov. (type strain CPCC 206450^T^=XZ100^T^=KCTC 59273^T^) were proposed. These strains exhibited stable growth under high-intensity UV radiation, attributed to the presence of *uvrABC* and *recAFNOQR* genes involved in UV resistance and DNA repair. These features indicate the *Knoellia* spp*.* adaptation to high UV radiation environments.

## Introduction

The Tibet Autonomous Region, situated atop the Qinghai–Tibet Plateau, is distinguished by its expansive high-altitude plateaus, cold and dry climate and intense solar radiation [1]. Typically, such extreme habitats are considered inhospitable to most life forms [2]. Nevertheless, micro-organisms inhabiting these environments have evolved specialized biochemical and physiological mechanisms enabling them to not only survive but also thrive under the harshest conditions [3]. For instance, *Planococcus halotolerans* Y50^T^, isolated from the Tibet Plateau, exhibits capabilities in petroleum degradation and can withstand high levels of oxidative stress, UV radiation and cold environments [4]. The presence of such microbes in extreme environments like the Tibetan Plateau offers a unique opportunity for the discovery of novel micro-organisms with distinctive properties, potentially unlocking new applications in biotechnology and environmental remediation.

The family *Intrasporangiaceae* was first described by Stackebrandt *et al*. [5] and subsequently emended by Stackebrandt and Schumann [6], Zhi *et al*. [7] and Nouioui *et al*. [8]. As of March 10, 2025, this family comprises 19 validly named genera (https://lpsn.dsmz.de/family/intrasporangiaceae). Notably, one genus, *Knoellia*, introduced by Groth *et al*. in 2002 with *Knoellia sinensis* as the type species [9], included seven species (https://lpsn.dsmz.de/genus/knoellia), which were obtained from diverse environments, including soil [9,10], pig manure [11] and air [12,13].

In this study, we isolated four *Knoellia* strains from the soil samples in Tibet Autonomous Region. Using polyphasic taxonomy and comparative genomics, we identified these strains as novel species within the genus *Knoellia*. Furthermore, our analyses revealed that these strains possess a high tolerance to UV radiation, as demonstrated by both genomic and UV tolerance assays.

## Methods

### Micro-organism acquisition

In our exploration of the diversity of actinobacteria on the Qinghai–Tibet Plateau, we collected soil samples from Nyingchi City, Shigatse City and Chamdo City in the Tibet Autonomous Region. After dilution of the sample using 0.85% (w/v) NaCl solution, ~200 µl of the 10^−4^ concentration soil suspension was plated onto the isolation media, respectively, and then incubated at 28 °C for 3 weeks to obtain distinct colonies. The distinct colonies were picked and streaked onto newly prepared Peptone-yeast-glycerol (PYG) medium plates (g l^−1^) (peptone 3, yeast extract 5, glycerol 10, betaine hydrochloride 1.25, sodium pyruvate 1.25, agar 15 and pH 7.2) to obtain pure cultures. The purified isolates were maintained as glycerol suspensions (20%, v/v) at −80 °C.

In this procedure, strain CPCC 206391^T^ (with the original number of XZ253^T^) covered on humic acid agar medium (g l^−1^) (humic acid 1, asparagine 1, FeSO_4_·7H_2_O 0.01, Na_2_HPO_4_·12H_2_O 0.5, KCl 1.7, CaCO_3_ 0.02, agar 15 and pH 7.2), from a soil sample collected at 3,026 m above sea level in Milin County, Nyingchi City (29° 37′ 13″ N 94° 24′ 12″ E); strain CPCC 206453^T^ (=CXZ644^T^), was recovered on cellulose agar medium (g l^−1^) (cellulose 2.5, proline 1, KNO_3_ 0.25, MgSO_4_·7H_2_O 0.2, K_2_HPO_4_ 0.2, CaCl_2_ 0.5, FeSO_4_·7H_2_O 0.01, betaine hydrochloride 2.5, sodium pyruvate 2.5, agar 15 and pH 7.2), from a sample collected at the altitude of 5,101 m in Nyalam County, Shigatse City (28° 30′ 49″ N 87° 4′ 13″ E); strain CPCC 206435^T^ (=CXZ904^T^) was recovered on chitin agar medium (g l^−1^) (chitin 2.0, K_2_HP0_4_ 0.7, KH_2_PO_4_ 0.3, MgSO_4_·7H_2_O 0.5, FeSO_4_ 0.1, sodium pyruvate 1.25, agar 15 and pH 7.2), from a sample collected at the altitude of 2,448 m in Zuogong County, Qamdo City (28° 45′ 56″ N 97° 28′ 18″ E); strain CPCC 206450^T^ (=XZ100^T^) appeared on humic acid agar plate, from a sample collected at the altitude of 3,789 m in Dingri County, Shigatse City (29° 25′ 39″ N 90° 48′ 52″ E).

Strains *Knoellia aerolata* JCM 16377^T^ and *Knoellia locipacati* JCM 17313^T^ obtained from the RIKEN BioResource Research Center (JCM) and strains *K. sinensis* KCTC 19790^T^, *Knoellia subterranea* KCTC 19937^T^ and *Knoellia flava* KCTC 19810^T^ obtained from the Korean Collection for Type Cultures (KCTC) were used as references for partial parallel experiments in this study.

### Phylogenetic analysis based on the 16S rRNA gene sequence

The 16S rRNA genes of these newly isolated strains were amplified by PCR using the universal bacterial primers 27F (5′-AGAGTTTGATCCTGGCTCAG-3′) and 1492R (5′-GGTTACCTTGTTACGACTT-3′). The purified PCR products were inserted into the pMD19-T vector (TaKaRa), and then, the recombinant plasmids were introduced into *Escherichia coli* DH5α cells. Subsequently, the plasmids were sequenced by Sangon Biotech (Shanghai, China). The blast program and the EzBioCloud (https://www.ezbiocloud.net/) [14] were used to compare the sequence of the isolates with available 16S rRNA gene sequences in GenBank to determine an approximate phylogenetic affiliation of the studied strain. Multiple sequence alignments of the most closely related taxa were conducted using mega version 11 [15]. A phylogenetic tree was then inferred using the neighbour-joining (NJ) method [16] with *K* values [17] and complete deletion gaps. Maximum parsimony [18] and maximum likelihood [19] phylogenetic methods were also used to evaluate the phylogenetic affiliations. The topologies of the resultant phylogenetic trees were evaluated using bootstrap analysis with 1,000 replicates [20].

### Genome sequencing, assembly, annotation and comparative genomic analysis

Genome sequencing was performed using the Illumina HiSeq 4000 system at the BGI sequencing company (Shenzhen, China). Genomic DNA was randomly fragmented to create three read libraries with 300 bp inserts, utilizing a Bioruptor ultrasonicator (Diagenode, Denville, NJ, USA) and physical–chemical methods. The paired-end libraries were then sequenced on the Illumina platform. Reads with low quality (defined as those with fewer than five reads covering consecutive bases) were excluded. The clean reads were subsequently assembled using the SOAPdenovo (version 1.05) software [21]. The completeness and contamination of assembled genomes were assessed using the CheckM pipeline [22]. Digital DNA–DNA hybridization (dDDH) and average nucleotide identity (ANI) values between these strains and their closely related type strains were calculated using the Genome-to-Genome Distance Calculator (version 3.0) [23] and the EzBioCloud platform [14], respectively. The genome-based phylogeny was constructed using a supermatrix approach with protein sequences from the bac 120 gene set (a set of 120 single-copy protein sequences commonly found in bacteria), employing EasyCGTree version 3.04 [24], as outlined in previous studies. The amino acid sequences of strains CPCC 206391^T^, CPCC 206453^T^, CPCC 206435^T^ and CPCC 206450^T^ were aligned against Kyoto Encyclopedia of Genes and Genomes [25] databases to obtain their corresponding annotations using eggnog Mapper v5.0 [26] with default options. The biosynthetic gene cluster of secondary metabolites was predicted through *in silico* analysis using antiSMASH 7.1.0 (https://antismash.secondarymetabolites.org/) [27].

### Growth conditions, physiological tests and morphological characteristic observation

The strains’ growth was tested on nutrient agar, tryptone soy agar (Difco), Reasoner’s 2A agar (R2A) (Difco) and PYG agar medium (PYG) at 28 °C for 48–72 h to check the optimal growth media. We cultivated strains at the temperature of 4, 10, 15, 20, 25, 28, 30, 32, 37 and 40 °C using PYG medium to test the growth temperature, as well as in the pH range of pH 5–10 (intervals of 1 pH units) and with NaCl concentration of 0–10% (intervals of 1%) (w/v) to test the growth condition in R2A broth medium. Oxidase activity was investigated using the API oxidase reagent (bioMérieux) according to the manufacturer’s instructions. Catalase activity was evaluated based on the production of bubbles with the addition of a drop of 3% (v/v) hydrogen peroxide. Metabolic characteristics were subsequently examined using Biolog GEN III (MicroPlate), API 50CH and API ZYM test kits (bioMérieux), according to the manufacturer’s instructions. Metabolic results were evaluated after incubation at 28 °C for 48–72 h. Other physiological tests, including the ability to produce H_2_S, gelatin liquefaction, starch hydrolysis and nitrate reduction, were conducted, as previously described [28]. The Gram reaction was tested by the standard Gram stain method [29], and the morphology of cells was observed by light microscopy (Zeiss Axio Scope, A1 Vario).

### Chemotaxonomic characterization

Chemotaxonomic characterization was conducted using cells collected from TSB medium cultivation in shake flasks on a rotary shaker (150 r.p.m.) at 28 °C to achieve logarithmic phase growth. Cellular polar lipids were then extracted, detected using two-dimensional TLC and identified according to the previously described procedures [30]. The respiratory quinones were extracted according to the method described by Minnikin *et al*. [30] and identified by HPLC [31]. Cellular fatty acids were extracted and analysed using the Sherlock Microbial Identification System (MIDI) according to the manufacturer’s instructions [32]. The MIDI Sherlock software program (version 6.0) and the TSBA 6 database were used for the analysis.

### The UV radiation resistance tests

The UV tolerance of these isolates was determined according to the procedures previously described [33], using the reference strain *E. coli* DH5α as a negative control. Briefly, strains in the logarithmic growth phase were inoculated onto PYG agar plates, with one group left unirradiated as the control, while other groups were exposed to different UV doses (120, 360, 600, 960 and 1440 J/m²), followed by incubation at 28 °C for 7 days. After the incubation period, the growth of the strains was evaluated and classified as either positive or negative in comparison to the unexposed control group.

## Results and discussion

### Strain identification based on the 16S rRNA gene sequences

**Fig. 1.**
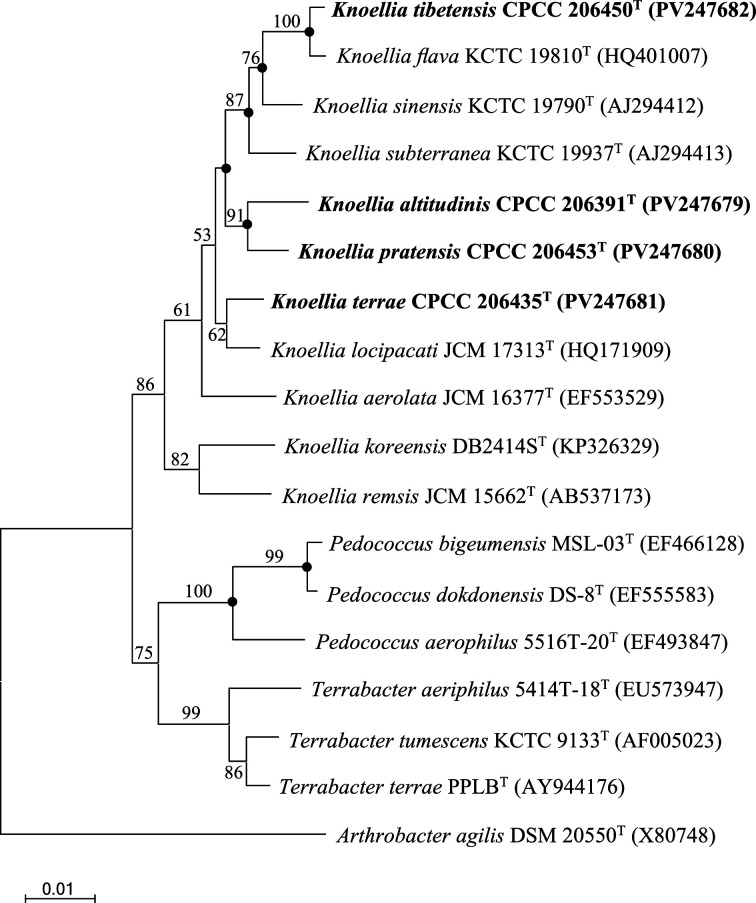
NJ tree based on 16S rRNA gene sequences showing the relationships of strains CPCC 206391^T^, CPCC 206435^T^, CPCC 206453^T^ and CPCC 206450^T^ with other representatives of the family *Intrasporangiaceae*. Filled circles indicate that the corresponding nodes were also recovered in phylogenetic trees generated using maximum likelihood and maximum parsimony methods. Bootstrap values above 50% are shown as percentages of 1,000 replicates. *Arthrobacter agilis* DSM 20550^T^ was used as the outgroup. Scale bar indicates 0.01 nt substitutions per alignment site.

Nearly complete 16S rRNA gene sequences for strains CPCC 206391^T^, CPCC 206435^T^, CPCC 206453^T^ and CPCC 206450^T^ were obtained. blast searches of the 16S rRNA gene sequences against the GenBank database indicated that strains CPCC 206391^T^, CPCC 206435^T^, CPCC 206453^T^ and CPCC 206450^T^ exhibited the highest similarities of 96.1–99.4% with *Knoellia* species of the family *Intrasporangiaceae*. These sequences were then compared pairwise with the 16S rRNA gene sequences of seven type strains of the validly described *Knoellia* species (Table S1, available in the online Supplementary Material). The 16S rRNA gene sequences of CPCC 206391^T^, CPCC 206453^T^ and CPCC 206435^T^ exhibited the highest similarities to *K. locipacati* JCM 17313^T^, with values of 97.5%, 98.4% and 98.9%, respectively. Strain CPCC 206450^T^ exhibited the highest similarity of 99.4% to *K. flava* KCTC 19810^T^. In phylogenetic trees reconstructed based on the 16S rRNA gene sequences (Figs 1, S1 and S2), strains CPCC 206391^T^ and CPCC 206453^T^, CPCC 206435^T^ and CPCC 206450^T^ clustered into the genus *Knoellia* clade, which suggested classifying these strains as members of the genus *Knoellia*.

### Whole-genome comparison and phylogenomics

The quality of the sequenced genomes was accurately assessed and deemed satisfactory using the CheckM pipeline (completeness >80% and contamination <5%, Table S2). The genomic G+C content of these four strains calculated from the draft genome sequence was as follows: 71.6% (CPCC 206391^T^), 69.9% (CPCC 206435^T^), 68.4% (CPCC 206453^T^) and 71.3% (CPCC 206450^T^). The ANI values calculated between strains CPCC 206391^T^, CPCC 206435^T^, CPCC 206453^T^, CPCC 206450^T^ and their closely related type strains of the genus *Knoellia* by comparison of their genomes [34] were all lower than the cutoff of 95–96% [35] (Table S3). Consistently, the dDDH values were found to be all below 70% [35] between strains CPCC 206391^T^, CPCC 206435^T^, CPCC 206453^T^, CPCC 206450^T^ and their related type strains of the genus *Knoellia* (Table S3). Notably, the ANI values between CPCC 206435^T^ and *K. flava* KCTC 19810^T^ were 91.5%, approaching the species threshold. However, a genomic nucleotide diversity of 10% represents tens of thousands of years of evolutionary divergence, providing robust evidence to classify them as distinct species [36,37]. High-throughput ANI analysis of 90,000 prokaryotic genomes reveals distinct species boundaries [35].

**Fig. 2.**
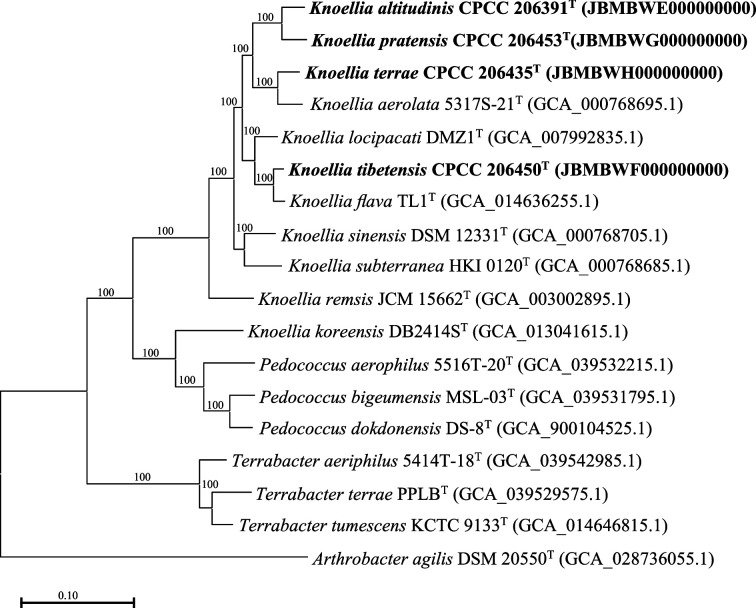
Genome-based phylogenetic tree (*Arthrobacter agilis* DSM 20550^T^ was used as the outgroup).

To further confirm the phylogenetic positions of these *Knoellia* strains, their genome sequences were determined and then subjected to phylogenomic tree reconstruction with other reference genomes (Fig. 2) (*Arthrobacter agilis* DSM 20550^T^ was used as the outgroup). Compared to 16S rRNA gene phylogeny, phylogenomics offered a more reliable phylogenetic topology. The strain CPCC 206435^T^ was clustered with *K. aerolata* JCM 16377^T^ with a bootstrap value of 100%. Strain CPCC 206450^T^ was clustered with *K. flava* KCTC 19810^T^ with a bootstrap value of 100% and *K. locipacati* JCM 17313^T^ to form a subclade. The phylogenetic placements of the strain CPCC 206391^T^ and CPCC 206453^T^ were utterly consistent with those in the 16S rRNA gene tree.

In conclusion, the overall genome-relatedness indices combined with phylogenetic analyses indicated that these four strains represent four novel genetic species of the genus *Knoellia*.

### Morphological, physiologic and biochemical characteristics

These four isolates obtained in this study were identified as Gram-stain-positive, non-motile, non-spore-forming, irregular rods and cocci. Strains CPCC 206391^T^, CPCC 206435^T^, CPCC 206453^T^ and CPCC 206450^T^ grew well on PYG and R2A agar. Colonies were circular, smooth, moist, translucent and convex after 2 days on PYG agar at 28 °C. The growth of all four strains was observed between 15 and 37 °C, with optimal growth at 28–30 °C for strains CPCC 206435^T^, CPCC 206453^T^ and CPCC 206450^T^, whereas strain CPCC 206391^T^ showed an optimal growth range of 25–32 °C. Strains CPCC 206453^T^ and CPCC 206450^T^ grew within the pH range of 6.0–8.0, with an optimal pH of 7.0, while strain CPCC 206391^T^ grew within the pH range of 6.0–9.0, with an optimal pH of 7.0. Strain CPCC 206435^T^, on the other hand, grew within the pH range of 6.0–7.0, with an optimum at pH 7.0. The highest NaCl tolerance was 4% (w/v) for strain CPCC 206391^T^ and CPCC 206450^T^ but only 1% (w/v) for strain CPCC 206435^T^ and CPCC 206453^T^. Strain CPCC 206391^T^ also grew well with no more than 2% (w/v) NaCl, while strain CPCC 206450^T^ could grow well with the presence of 4% (w/v) NaCl.

The key phenotypic characteristics of these four isolates were compared with those of their closely related species in terms of phenotypic analysis in Table 1. Strain CPCC 206450^T^ could be differentiated from *K. flava* KCTC 19810^T^ by their lack of trypsin, *α*-glucosidase and *β*-glucosidase activities but positive for hydrolysis of gelatin and starch. Strain CPCC 206435^T^ could be differentiated from *K. aerolata* JCM 16377^T^ by its positive activities of cystine aminopeptidase, trypsin and acid phosphatase but negative for hydrolysis of gelatin and starch and utilization of d-cellobiose and gluconate. Differentials between CPCC 206391^T^ and CPCC 206453^T^ were activities of lipase (C14) and *β*-galactosidase, moreover, and the assimilation of some carbon sources.

**Table 1.** Differentiating characteristics between the newly isolated strains and the related *Knoellia* reference strains Strains: 1, CPCC 206391^T^; 2, CPCC 206453^T^; 3, CPCC 206435^T^; 4, CPCC 206450^T^; 5, *K. aerolata* JCM 16377^T^; 6, *K. flava* KCTC 19810^T^; 7, *K. locipacati* JCM 17313^T^; 8, *K. sinensis* KCTC 19790^T^; 9, *K. subterranea* KCTC 19937^T^. The data for strains *K. locipacati* JCM 17313^T^ and *K. aerolata* JCM 16377^T^ were obtained from the Weon *et al*. [13] and Shin *et al*. [10]. All other data were taken from this study. +, Positive; w, weakly positive; −, negative activities/growth; nd, no data.

Characteristic	1	**2**	3	4	5	6	7	8	9
Optimum growth temperature (℃)	25–32	28–30	28–30	28–30	30	28–30	30	25–32	25–32
pH range with growth	6–9	6–7	6–8	6–8	6–7	6–7	7–8	6–9	6–9
NaCl tolerance (%, w/v)	0–4	0–1	0–1	0–4	0–2	0–4	0–5	0–4	0–5
Hydrolysis of gelatin	_	_	_	+	+	_	nd	_	_
Starch hydrolysis	+	+	_	+	+	_	+	+	w
Enzyme activities									
Lipase (C14)	+	−	−	−	nd	−	nd	−	−
Cystine aminopeptidase	w	+	+	+	−	+	−	+	+
Trypsin	w	w	+	−	−	+	w	w	+
Acid phosphatase	w	+	+	−	−	w	+	−	w
Naphthol-AS BI-phosphohydrola	+	+	+	+	−	+	+	+	+
*β*-Galactosidase	−	+	−	−	+	−	w	−	−
*β*-Glucuronidase	−	−	+	−	+	−	−	−	−
*α*-Glucosidase	+	+	+	−	−	+	+	+	w
*β*-Glucosidase	+	+	+	−	+	+	+	+	+
**Utilization of**									
Gentiobiose	−	w	+	−	w	+	w	−	−
d-Melibiose	−	w	w	+	nd	w	nd	−	−
d-Mannose	−	+	w	+	+	+	+	−	−
d-Galactose	−	+	w	+	w	−	+	−	+
*γ*-Amino-butryric acid	−	w	−	−	nd	+	nd	−	−
*α*-Hydroxy butyric acid	−	+	−	−	nd	w	nd	+	+
*α*-Keto-butyric acid	−	+	−	−	nd	−	nd	w	+
Glycerol	−	+	−	−	−	−	−	−	−
Methyl *α*-d-mannopyranoside	−	+	−	−	w	−	w	−	−
d-cellobiose	−	−	−	+	+	w	+	w	w
Inulin	−	+	−	w	+	w	−	−	−
d-Melezitose	−	+	−	−	+	w	w	−	−
d-Raffinose	−	+	−	−	w	w	−	−	−
Glycogen	−	+	−	−	nd	+	nd	−	w
Gentiobiose	−	+	−	−	w	w	w	−	−
d-Turanose	−	+	+	−	+	−	+	−	w
Gluconate	−	w	−	+	+	−	−	−	−
5-Ketogluconate	w	−	w	−	−	+	w	−	−
DNA G+C content (%)	69.9	68.4	71.3	71.6	73.0	71.5	72.6	68.3	69.0
Major fatty acids (>10%)	iso-C_15:0_ iso-C_16:0_ C_17:1_* ω*8*c*	iso-C_15:0_, iso-C_16:0_, C_17:0_ 10-methyl Summed Feature 9*	iso-C_15:0_ iso-C_16:0_ C_17:1_* ω*8*c* C_18:1_* ω*9*c*	iso-C_15:0_ iso-C_16:0_ C_17:1_* ω*8*c*	iso-C_15:0_ iso-C_16:0_ C_17:1_* ω*8*c*	iso-C_15:0_ iso-C_16:0_	iso-C_14:0_ iso-C_15:0_ iso-C_16:0_	iso-C_15:0_ iso-C_16:0_ Summed feature 9*	iso-C_14:0_ iso-C_15:0_ iso-C_16:0_

*Summed feature 9 contains iso-C_17:1_* ω*9*c* and/or C_16:0_ 10-methyl.

### Chemotaxonomic properties

The major polar lipids of four strains were phosphatidylinositol (PI), phosphatidylethanolamine (PE) and diphosphatidylglycerol (DPG) (Fig. S3), which is consistent with their related species and most of the other *Knoellia* species [9]. Differentially, phosphatidylglycerol was detected in the strains 5317 S-21^T^ and DMZ1^T^ but not in four strains isolated from this study. The predominant cellular fatty acids in all four strains were iso-C_16:0_ and iso-C_15:0_, which align with the fatty acid composition found in other members of the genus *Knoellia* [9,12]. Their fatty acid profiles could differentiate them from each other (Table S4). All of the tested strains contained the MK-8(H_4_) as the predominant respiratory quinone.

### UV radiation resistance

After exposure to UV radiation at the set doses, the growth of the four strains weakened with increasing exposure dose, but all showed higher UV resistance compared to *E. coli* DH5α. Notably, CPCC 206450^T^ survived at an intensity of 1,440 J m^−^² of UV exposure, while strains CPCC 206453^T^ and CPCC 206435^T^ exhibited growth at 960 J m^−^² of UV exposure. Strain CPCC 206391^T^ could only tolerate UV radiation up to 600 Jm^−^² of UV exposure.

**Fig. 3.**
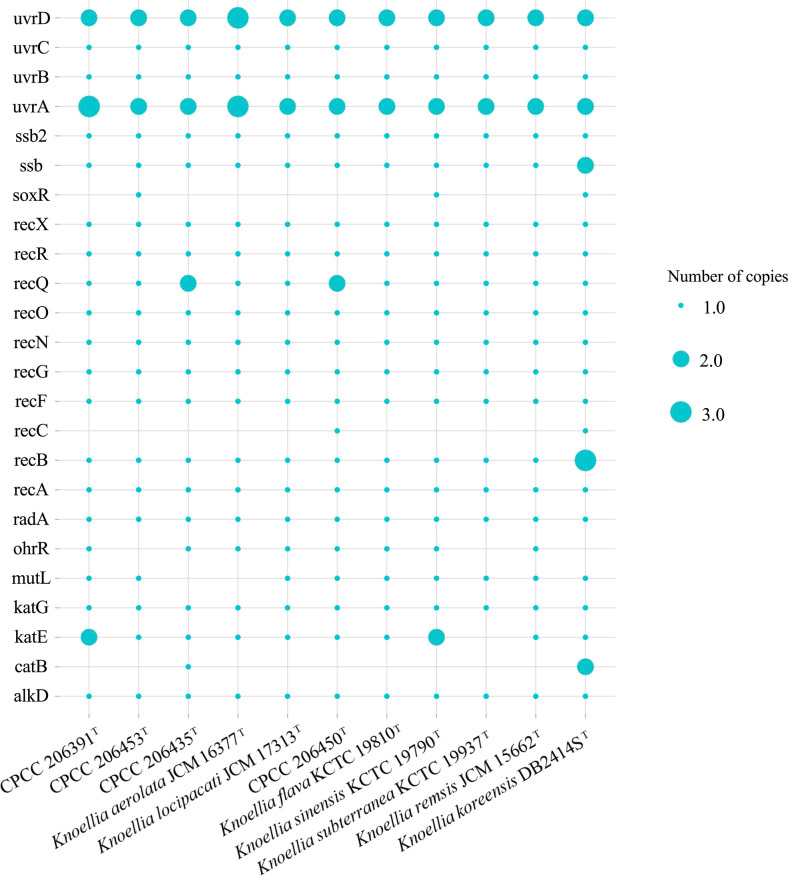
UV radiation DNA repair response genes in strain genomes.

DNA repair mechanisms are deemed to be the most important repair systems for radiation resistance [38]. Notably, the DNA repair genes associated with radiation resistance with several numbers of gene copies were detected in all of the type strains in genus *Knoellia* (Fig. 3). All of the type strains in *Knoellia* harbour the cascade of *recAFNOQR*, which participates in DNA repair and recombination [39], and the *uvrABC* pathway, which plays a significant role in the nucleotide excision repair (NER) [40]. The alkylation DNA repair dioxygenase *alkD* [41], which removes positively charged methylpurines from DNA and adopts a protein fold distinct from those of other DNA repair proteins, along with *mutL* [42], which mediates protein–protein interactions during mismatch recognition, was also identified in the genome of these strains. Moreover, most of the *Knoellia* strains possess the *radA* recombinase which is involved in processing recombination intermediates, stimulating branch migration of RecA-mediated strand transfer reactions and repairing DNA breaks [43]. Among the DNA repair genes, the *ssb* gene was also identified, which may bind and repair broken single-stranded DNA during the early stages of damage repair [44].

These observations suggested that the *Knoellia* spp. may have the capability to be resistant to high-level UV on a genetic basis. In addition, biosynthetic gene cluster of alkylresorcinol, which can improve DNA damage repair capacity by transitioning linearized DNA molecules from supercoiled to relaxed and from relaxed to linearized forms [45], was detected in the genome of these novel strains (CPCC 206453^T^, CPCC 206435^T^, CPCC 206391^T^ and CPCC 206450^T^), highlighting their capability of high-level UV resistance (Table S5). Combined with the assay of UV resistance, these four novel strains could survive in the high-level UV radiation ecosystems through multiple DNA repair systems.

## Description of *Knoellia altitudinis* sp. nov.

*Knoellia altitudinis* (al.ti.tu’di.nis. L. gen. n. *altitudinis*, of a high place).

Cells are Gram-stain-positive, aerobic, non-motile, non-spore-forming, irregular rods (0.6×1.0–1.9 µm) and cocci (0.5–0.9 µm diameter). Colonies are yellow, circular, smooth, translucent and convex (0.5–1.0 mm diameter) after 2 days on PYG agar at 28 ℃. Growth occurs at 15–37 ℃ (optimum 25–32 ℃), at pH 6–9 (optimum pH 7) and with 0–4% NaCl (optimum 1%). Negative for the catalase reaction, oxidase reaction, gelatin liquefaction, nitrate reduction and H_2_S production assays but weakly positive for starch hydrolysis. With API 50 CH, assimilates aesculin ferric citrate. With API ZYM, positive for alkaline phosphatase, esterase (C4), esterase lipase (C8), lipase (C14), leucine aminopeptidase, phosphohydrolase, valine aminopeptidase, *α*-glucosidase and *β*-glucosidase. According to results from Biolog GEN III test strips acetoacetic acid, acetic acid, d-maltose, d-trehalose, d-cellobiose, d-turanose, d-raffinose, d-glucose-6-PO_4_, d-fructose, d-fucose, d-salicin, d-sorbitol, d-mannitol, d-fructose-6-PO_4_, d-arabitol, d-gluconic acid, glucuronamide, glycerol, inosine, l-fucose, l-aspartic acid, l-glutamic acid, l-histidine, pectin, l-malic acid, myo-inositol, *N*-acetyl-d-glucosamine, *N*-acetyl-d-galactosamine, *N*-acetyl-*β*-d-mannosamine, *α*-d-glucose, propionic, stachyose, sucrose, Tween 40 and *α*-keto-butyric acid used for growth. The main cellular fatty acids are iso-C_16:0_, iso-C_15:0_ and C_17:1_* ω*8*c*. The major polar lipids are DPG, PI and PE. The predominant isoprenoid quinone is MK-8(H_4_).

The type strain CPCC 206391^T^ (=XZ253^T^=KCTC 59139^T^) was isolated from a soil sample collected from Milin County, Nyingchi City, Tibet Autonomous Region, China. It has a genome size of 3.82 Mb and DNA G+C content of 69.9%. The GenBank accession numbers of the 16S rRNA gene sequence and genome are PV247679 and JBMBWE000000000, respectively.

## Description of *Knoellia tibetensis* sp. nov.

*Knoellia tibetensis* (ti.bet.en’sis. N.L. fem. adj. *tibetensis,* pertaining to Tibet, an autonomous region of China).

Cells are Gram-stain-positive, aerobic, non-motile, non-spore-forming, irregular rods (0.6×1.0–1.9 µm) and cocci (0.5–0.9 µm diameter). Colonies are yellow, circular, smooth, translucent and convex (0.5–1.0 mm diameter) after 2 days on PYG agar at 28 ℃. Growth occurs at 15–37 ℃ (optimum 28–30 ℃), at pH 6–8 (optimum pH 7) and with 0–4% NaCl (optimum 1%). Negative for the catalase reaction, oxidase reaction, starch hydrolysis, gelatin liquefaction, nitrate reduction and H_2_S production assays but positive for starch hydrolysis and gelatin liquefaction. With API 50 CH, assimilates arbutin, d-cellobiose, aesculin ferric citrate and gluconate. With API ZYM, positive for alkaline phosphatase, cystine aminopeptidase, esterase (C4), esterase lipase (C8), leucine aminopeptidase, phosphohydrolase and valine aminopeptidase. According to results from Biolog GEN III test strips, acetoacetic acid, acetic acid, bromo-succinic acid, d-gluconic acid, d-saccharic acid, d-glucuronic acid, d-malic acid, d-lactic acid methyl ester, dextrin, d-maltose, d-trehalose, d-cellobiose, d-turanose, d-raffinose, d-melibiose, d-salicin, d-mannose, d-fructose, d-galactose, d-fucose, d-glucose-6-PO_4_, d-galacturonic acid, d-fructose-6-PO_4_, d-aspartic acid, *α*-d-glucose, *α*-d-lactose, formic acid, glycerol, gelatin, glycyl-l-proline, inosine, myo-inositol, l-alanine, l-lactic acid, l-arginine, l-aspartic acid, l-glutamic acid, l-histidine, l-pyroglutamic acid, l-serine, l-malic acid, l-butyric acid, l-galactonic acid lactone, mucic acid, methyl pyruvate, *N*-acetyl-d-glucosamine, *N*-acetyl-*β*-d-mannosamine, *N*-acetyl-d-galactosamine, *N*-acetyl neuraminic acid, pectin, quinic acid, sucrose, stachyose, *α*-keto-glutaric acid, *β*-methyl-d-glucoside, *β*-hydroxy-d and Tween 40 used for growth. The main cellular fatty acids are iso-C_16:0_, iso-C_15:0_ and C_17:1_* ω*8*c*. The major polar lipids are PI, PE and DPG. The predominant isoprenoid quinone is MK-8(H_4_).

The type strain CPCC 206450^T^ (=XZ100^T^=KCTC 59273^T^) was isolated from a soil sample collected from Dingri County, Shigatse City, Tibet Autonomous Region, China. It has a genome size of 3.77 Mb and DNA G+C content of 71.6%. The GenBank accession numbers of the 16S rRNA gene sequence and genome are PV247682 and JBMBWF000000000, respectively.

## Description of *Knoellia terrae* sp. nov.

*Knoellia terrae* (ter’rae. L. gen. n. *terrae*, of the earth, referring to the organism being isolated from soil).

Cells are Gram-stain-positive, aerobic, non-motile, non-spore-forming, irregular rods (0.6×1.0–1.9 µm) and cocci (0.5–0.9 µm diameter). Colonies are yellow, circular, smooth, translucent and convex (0.5–1.0 mm diameter) after 2 days on PYG agar at 28 ℃. Growth occurs at 15–37 ℃ (optimum 28–30 ℃), at pH 6–7 (optimum pH 7) and with 0–1% NaCl. Negative for the catalase reaction, oxidase reaction, gelatin liquefaction, nitrate reduction and H_2_S production assays but positive for starch hydrolysis. With API 50 CH, assimilates d-turanose, aesculin ferric citrate, 2-ketogluconate, 5-ketogluconate and starch. With API ZYM, positive for acid phosphatase, cystine aminopeptidase, esterase (C4), esterase lipase (C8), leucine aminopeptidase, phosphohydrolase, phosphohydrolase, trypsin, *α*-glucosidase, *β*-glucuronidase and *β*-glucosidase. According to results from Biolog GEN III test strips, acetoacetic acid, acetic acid, d-galacturonic acid, d-gluconic acid, d-glucose-6-PO_4_, d-fructose-6-PO_4_, d-aspartic acid, d-saccharic acid, d-malic acid, d-glucuronic acid, d-maltose, d-trehalose, d-turanose, stachyose, d-raffinose, *α*-d-lactose, d-melibiose, d-mannose, d-fructose, d-galactose, d-fucose, *β*-methyl-d-glucoside, d-salicin, α-d-glucose, d-sorbitol, d-mannitol, d-arabitol, gentiobiose, glycerol, glycyl-l-proline, glucuronamide, l-fucose, l-rhamnose, l-aspartic acid, l-glutamic acid, l-pyroglutamic acid, l-serine, l-galactonic acid lactone, l-butyric acid, l-malic acid, mucic acid, myo-inositol, *N*-acetyl-d-glucosamine, *N*-acetyl-*β*-d-mannosamine, quinic acid, *β*-hydroxy-d, pectin and sucrose used for growth. The main cellular fatty acids are iso-C_16:0_, iso-C_15:0_, C_17:1_* ω*8*c* and C_18:1_* ω*9*c*. The major polar lipids are DPG, PI and PE. The predominant isoprenoid quinone is MK-8(H_4_).

The type strain CPCC 206435^T^ (=CXZ904^T^=KCTC 59271^T^) was isolated from a soil sample collected from Zuogong County, Qamdo City, Tibet Autonomous Region, China. It has a genome size of 3.47 Mb and DNA G+C content of 71.3%. The GenBank accession numbers of the 16S rRNA gene sequence and genome are PV247681 and JBMBWH000000000, respectively.

## Description of *Knoellia pratensis* sp. nov.

*Knoellia pratensis* (pra.ten’sis. L. fem. adj. *pratensis*, found in meadows/grassland).

Cells are Gram-stain-positive, aerobic, non-motile, non-spore-forming, irregular rods (0.6×1.0–1.9 µm) and cocci (0.5–0.9 µm diameter). Colonies are yellow, circular, smooth, translucent and convex (0.5–1.0 mm diameter) after 2 days on PYG agar at 28 ℃. Growth occurs at 15–37 ℃ (optimum 28–30 ℃), at pH 6–8 (optimum pH 7) and with 0–1% NaCl. Negative for the catalase reaction, oxidase reaction, gelatin liquefaction, nitrate reduction and H_2_S production assays and starch hydrolysis. With API 50 CH, assimilates amygdalin, d-ribose, methyl *α*-d-mannopyranoside, d-melibiose, d-sucrose, d-trehalose, d-melezitose, d-raffinose, d-turanose, d-tagatose, d-fucose, d-arabitol, aesculin ferric citrate, glycerol, glycogen, gluconate, gentiobiose, inulin, l-fucose, l-rhamnose, l-arabitol, 2-ketogluconate, xylitol and starch. With API ZYM, positive for alkaline phosphatase, acid phosphatase, cystine aminopeptidase, esterase (C4), esterase lipase (C8), *β*-galactosidase, *α*-glucosidase, *β*-glucosidase, leucine aminopeptidase, phosphohydrolase, trypsin and valine aminopeptidase. According to results from Biolog GEN III test strips, acetoacetic acid, acetic acid, bromo-succinic acid, d-maltose, d-trehalose, d-cellobiose, d-turanose, d-melibiose, *α*-d-glucose, d-mannose, d-fructose, d-galactose, d-fucose, d-sorbitol, d-mannitol, d-arabitol, d-fructose-6-PO_4_, d-gluconic acid, d-lactic acid methyl ester, gentiobiose, inosine, l-aspartic acid, l-fucose, l-rhamnose, l-glutamic acid, l-butyric acid, l-histidine, l-pyroglutamic acid, l-lactic acid, l-malic acid, *N*-acetyl-d-glucosamine, *N*-acetyl-*β*-d-mannosamine, *N*-acetyl-d-galactosamine, propionic acid, sucrose, Tween 40, *α*-hydroxybutyric acid, *α*-keto-butyric acid, *β*-hydroxy-d and *γ*-amino-butryric acid used for growth. The main cellular fatty acids are iso-C_16:0_, iso-C_15:0_, C_17:0_ 10-methyl and summed feature 9 (contains iso-C_17:1_* *ω*9c* and/or C_16:0_ 10-methyl). The major polar lipids are DPG, PI and PE. The predominant isoprenoid quinone is MK-8(H_4_).

The type strain CPCC 206453^T^ (=CXZ644^T^=KCTC 59274^T^) was isolated from a soil sample collected from Nyalam County, Shigatse City, Tibet Autonomous Region, China. It has a genome size of 4.09 Mb and DNA G+C content of 68.4%. The GenBank accession numbers of the 16S rRNA gene sequence and genome are PV247680 and JBMBWG000000000, respectively.

## Supplementary material

10.1099/ijsem.0.006855 Uncited Supplementary Material 1.

## Abbreviations

ANI: average nucleotide identity
dDDH: digital DNA–DNA hybridization
DPG: diphosphatidylglycerol
KCTC: Korean Collection for Type Cultures
NJ: neighbour-joining
PE: phosphatidylethanolamine
PI: phosphatidylinositol
R2A: Reasoner’s 2A agar
