# Supplementary material for: Knoellia altitudinis sp. nov., Knoellia pratensis sp. nov., Knoellia terrae sp. nov. and Knoellia tibetensis sp. nov., four novel UV radiation-resistant actinobacteria isolated from Tibet Autonomous Region, China
Source: Int J Syst Evol Microbiol. 2025 Aug 8;75(8):006855. doi: 10.1099/ijsem.0.006855 (PMC12451619; doi:10.1099/ijsem.0.006855)
Supplement: Uncited Supplementary Material 1. [file ijsem-75-06855-s001.pdf]

Knoellia altitudinis sp. nov., _Knoellia pratensis_ sp. nov., _Knoellia terrae_ sp. nov. and _Knoellia tibetensis_ sp. nov., four novel UV radiation resistance actinobacteria isolated from Tibet Autonomous Region, China

Jing Zhang[1], Tong Mou[1], Cong-Jian Li[1], Jing-Lin Bai[1], Li-Yan Yu[1], Hua-Hong Chen[2], Yu-Qin Zhang[1*]

[1]Institute of Medicinal Biotechnology, Chinese Academy of Medical Sciences & Peking Union Medical College, Beijing 100050, P. R. China

[2]College of Resources, Environmental Sciences and Chemistry, Chuxiong Normal University, Chuxiong, Yunnan, 675000, P. R. China

[*]Author for correspondence:

Yu-Qin Zhang

Tel: +86-10-83167110

Fax: +86-10-83167110

E-Mail: yzhang@imb.pumc.edu.cn

Supplementary Figure S1. Maximum-likelihood tree based on 16S rRNA gene sequences showing the relationships of strains CPCC 206391^T, CPCC 206435^T, CPCC 206453^T and CPCC 206450^T with other representatives of the family *Intrasporangiaceae*. Bootstrap values above 50% are shown as percentages of 1,000 replicates. *Arthrobacter agilis* DSM 20550^T was used as the outgroup. Scale bar indicates 0.05 nt substitutions per alignment site.

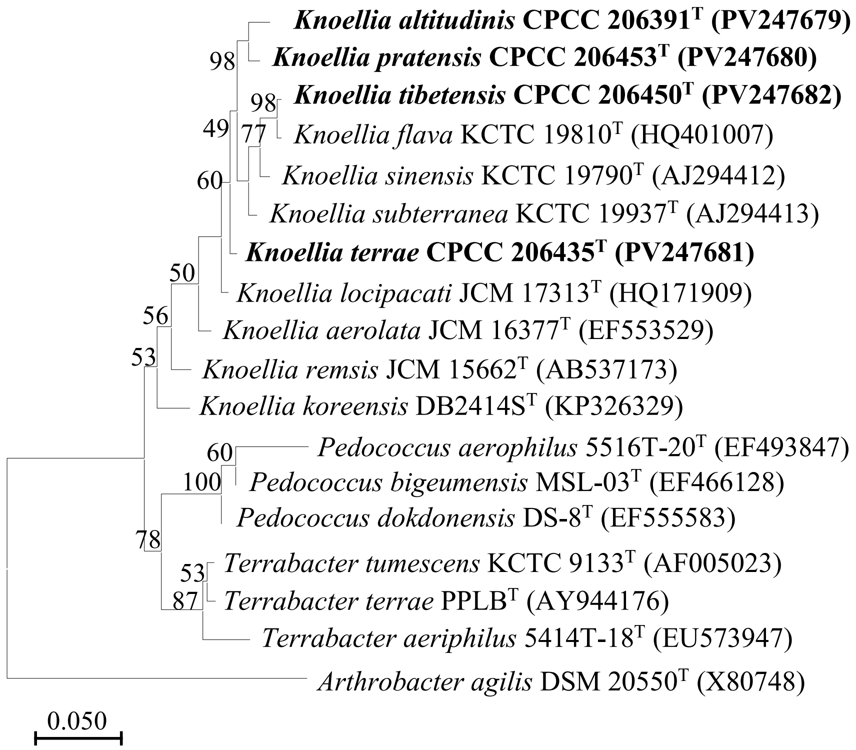

Supplementary Figure S2. Maximum Parsimony tree based on 16S rRNA gene sequences showing the relationships of strains CPCC 206391^T, CPCC 206435^T, CPCC 206453^T and CPCC 206450^T with other representatives of the family *Intrasporangiaceae*. Bootstrap values above 50% are shown as percentages of 1,000 replicates. *Arthrobacter agilis* DSM 20550^T was used as the outgroup.

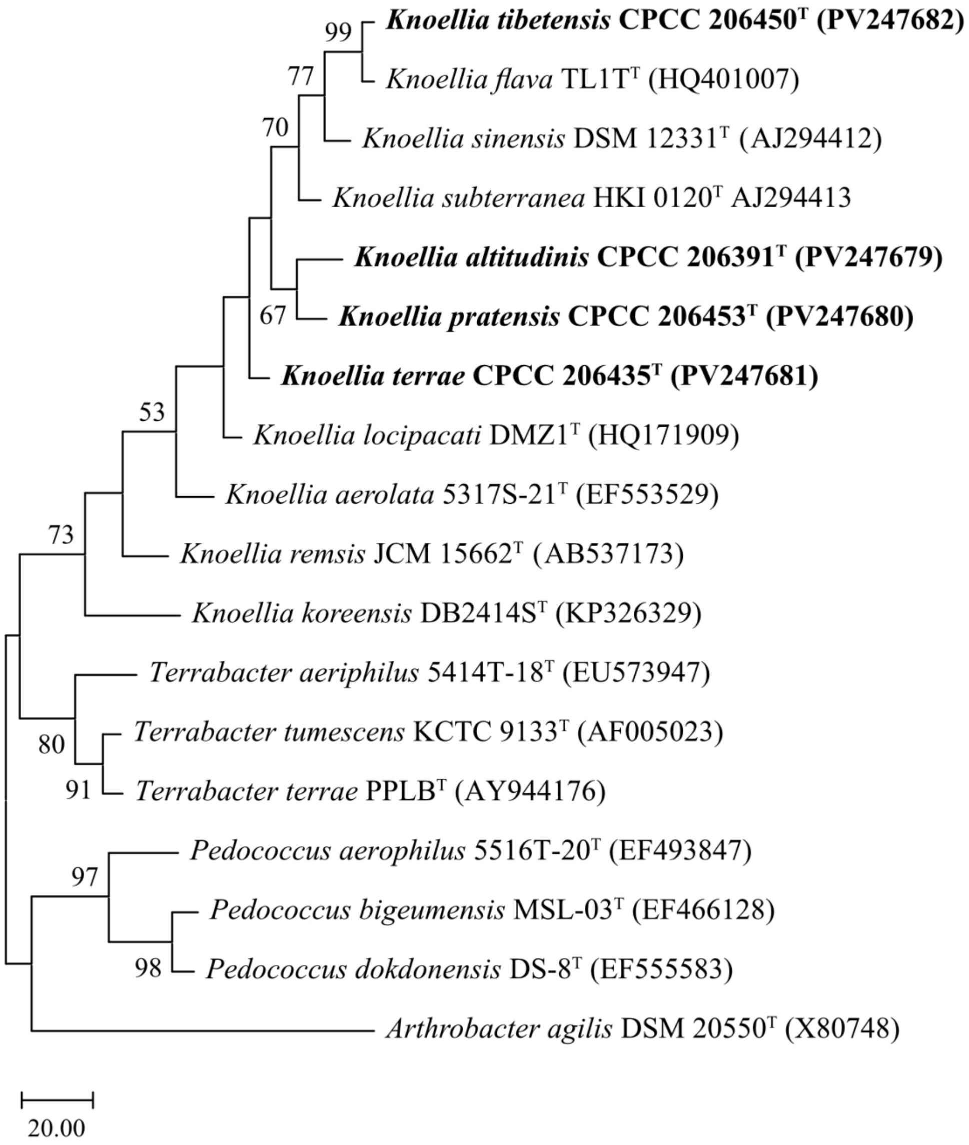

Supplementary Figure S3. Polar lipid profiles of strains CPCC 206391^{T}, CPCC 206435^{T}, CPCC 206453^{T}, CPCC 206450^{T} and other type strains of members of the genus *Knoellia* after separation by two-dimensional thin layer chromatography.

(a) was detected by spraying with molybdatophosphoric acid reagent; (b) was detected by spraying with molybdenum blue stain reagent; (c) was detected by spraying with ninhydrin stain reagent. DPG, diphosphatidylglycerol; PE, phosphatidylethanolamine; PI, phosphatidylinositol.

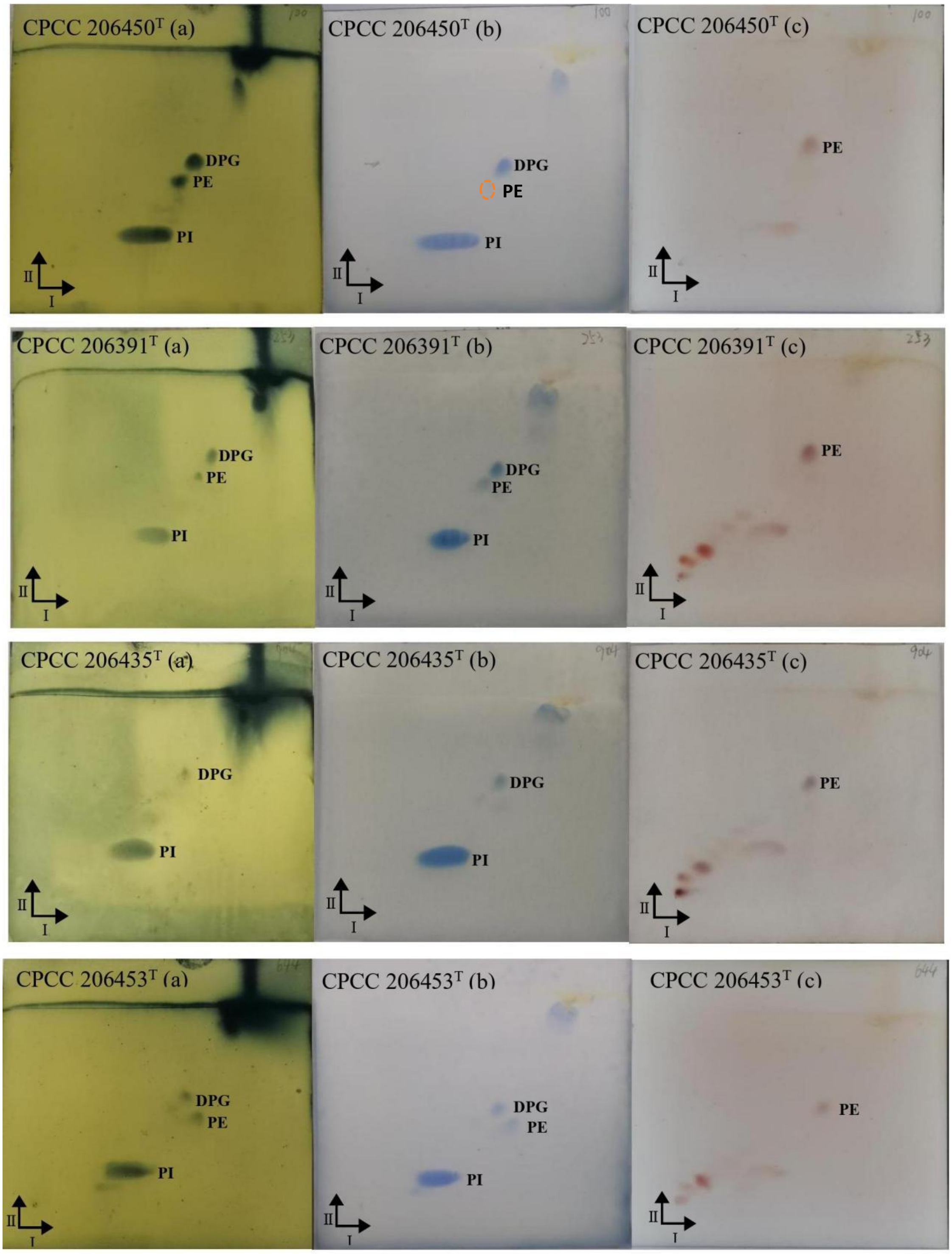

Supplementary table S1. The pairwise 16S rRNA gene sequence similarity between strains CPCC 206391^T, CPCC 206435^T, CPCC 206453^T, CPCC 206450^T and the 7 validly named *Knoellia* species.

Notes: 1, CPCC 206391^T; 2, CPCC 206453^T; 3, CPCC 206435^T; 4, CPCC 206450^T; 5, *K. sinensis*

Strain	16S rRNA gene sequence similarity(%)										
	1	2	3	4	5	6	7	8	9	10	11
1	**100.0**										
2	98.2	**100.0**									
3	97.7	98.2	**100.0**								
4	96.9	97.7	97.6	**100.0**							
5	97.3	97.8	98.2	98.6	**100.0**						
6	97.5	98.2	98.3	98.3	98.5	**100.0**					
7	96.5	97.4	98.3	99.4	98.5	98.1	**100.0**				
8	96.1	96.9	96.6	96.4	96.5	96.4	96.2	**100.0**			
9	96.8	97.3	97.9	97.2	97.2	97.1	96.9	96.9	**100.0**		
10	96.2	96.7	97.0	97.6	97.3	96.5	97.4	97.9	97.5	**100.0**	
11	97.5	98.4	98.9	98.0	98.3	98.3	98.0	97.6	97.9	97.5	**100.0**

KCTC 19790^T; 6, *K. subterranea* KCTC 19937^T; 7, *K. flava* KCTC 19810^T; 8, *K. koreensis* DB2414S^T; 9, *K. aerolata* JCM 16377^T; 10, *K. remsis* JCM 15662^T; 11, *K. locipacati* JCM 17313^T.

Supplementary table S2. CheckM pipeline assessment of genome quality of strains strains CPCC 206391^T, CPCC 206435^T, CPCC 206453^T and CPCC 206450^T.

Strain	Completeness(%)	Contamination(%)
CPCC206391^T	100	0
CPCC206435^T	100	0
CPCC206450^T	99.73	0
CPCC206453^T	100	0.54

Supplementary table S3. Average nucleotide identity (ANI) values (lower triangle) and digital DNA-DNA hybridization (dDDH) values (up triangle) between strains CPCC 206391^T, CPCC 206435^T, CPCC 206453^T, CPCC 206450^T and related strains of the genus *Knoellia*.

Strain	1	2	3	4	5	6	7	8	9	10	11
1		24.7	24.7	28.0	25.4	24.9	19.8	25.4	23.2	23.7	23.9
2	84.2		25.6	24.0	31.4	25.7	20.3	26.0	23.7	23.4	23.4
3	84.4	85.0		24.4	25.9	41.0	20.5	30.2	24.4	24.4	24.6
4	85.7	82.7	83.2		24.3	24.0	20.5	30.2	24.4	24.4	24.6
5	83.7	87.2	84.6	82.7		25.7	20.1	26.5	24.0	23.7	23.9
6	84.3	85.1	91.5	83.4	85.1		20.4	30.1	24.5	24.3	24.8
7	78.5	78.8	79.1	78.1	78.8	79.2		20.6	21.3	20.1	20.1
8	84.8	85.2	87.7	84.2	85.3	87.8	79.5		24.4	24.6	24.8
9	82.8	83.1	83.8	82.0	83.1	84.0	79.8	83.8		22.8	23.3
10	82.4	82.2	82.9	82.5	82.0	83.1	77.7	83.2	81.6		24.0
11	83.3	82.8	84.0	83.3	82.8	84.1	78.4	84.2	82.7	83.4	

Notes: 1, CPCC 206391^T ;2, CPCC 206435^T; 3, CPCC 206450^T; 4, CPCC 206453^T; 5, *K. aerolata* JCM 16377^T; 6, *K. flava* KCTC 19810^T; 7, *K. koreensis* DB2414S^T; 8, *K. locipacati* JCM 17313^T; 9, *K. remsis* JCM 15662^T; 10, *K. sinensis* KCTC 19790^T; 11, *K. subterranea* KCTC 19937^T.

Supplementary table S4. Cellular fatty acid profiles of strains CPCC 206391^T, CPCC 206435^T, CPCC 206453^T, CPCC 206450^T and other type strains of the genus *Knoellia*.

Fatty acid	1	2	3	4	5	6	7	8	9
iso-$C_{12:0}$	-	-	-	0.8	ND	-	ND	0.5	-
iso-$C_{13:0}$	TR	-	0.5	1.1	TR	-	ND	1.5	-
iso-$C_{14:0}$	3.8	2.7	7.2	3.8	4.5	6.3	10.6	10.1	5.4
$C_{14:0}$	TR	0.6	TR	2.0	5.4	0.9	TR	0.7	-
iso-$C_{15:0}$	13.4	21.3	20.5	12.1	10.5	14.5	12.6	21.9	17.9
anteiso-$C_{15:0}$	1.3	1.8	1.4	1.1	ND	0.7	ND	5.1	1.2
$C_{15:1}$ $\omega 6c$	TR	-	1.2	TR	ND	-	ND	1.2	-
$C_{15:1}$ $\omega 8c$	-	0.7	-	0.5	ND	-	ND	TR	-
$C_{15:0}$ -3-OH	-	2.1	-	-	ND	-	ND	-	-
iso-$C_{15:0}$-3-OH	-	-	-	TR	ND	-	ND	1.2	-
iso-$C_{16:0}$-3-OH	-	-	-	-	ND	-	ND	1.3	-
iso-$C_{16:1}$ -H	3.4	0.9	0.8	1.5	ND	5.0	1.1	1.6	2.9
iso-$C_{16:0}$	23.1	12.3	17.3	10.8	9.2	29.6	32.6	18.3	29.0
$C_{16:0}$	1.5	2.0	2.5	2.8	14.4	1.5	1.4	1.2	2.2
$C_{16:1}$ 2-OH	2.2	-	-	-	ND	-	ND	1.1	0.9
anteiso-$C_{17:1}$ $\omega 9c$	0.7	-	-	0.7	ND	-	ND	0.8	0.7
iso-$C_{17:0}$	2.2	3.1	1.2	1.8	ND	2.4	ND	3.9	4.0
anteiso-$C_{17:0}$	1.5	2.4	0.7	1.9	ND	1.0	ND	2.4	1.4
$C_{17:1}$ $\omega 8c$	9.0	14.7	21.1	17.2	11.3	9.9	9.4	3.9	1.6
$C_{17:1}$ $\omega 6c$	-	-	-	-	ND	-	ND	-	2.1
$C_{17:0}$	1.8	3.1	5.0	2.3	11.5	1.3	6.1	0.9	2.6
$C_{17:0}$ -3-OH	TR	-	-	-	ND	1.91	4.1	TR	-
10-methyl $C_{17:0}$	13.9	9.9	4.3	2.1	ND	9.6	9.8	4.4	4.4
iso-$C_{18:0}$	1.3	-	-	0.5	ND	1.4	ND	0.6	0.6
$C_{18:1}$ $\omega 9c$	1.2	4.7	6.1	14.5	5.4	3.0	TR	0.7	-
$C_{18:3}$ $\omega 6c$ (6,9,12)	-	1.1	-	TR	ND	-	ND	-	-
$C_{18:0}$	0.6	1.1	0.72	1.8	5.4	1.4	ND	TR	-
$C_{18:0}$ 10-methyl, TBSA	-	3.0	-	-	ND	-	ND	0.6	-
Summed Feature 3*	2.7	1.1	3.2	6.3	ND	2.8	ND	3.5	1.5
Summed Feature 6*	1.0	2.3	0.6	2.3	ND	-	ND	0.5	0.8
Summed Feature 9*	12.9	7.1	2.9	5.7	ND	3.7	ND	7.3	17.8

Notes: 1, CPCC 206453^T; 2, CPCC 206450^T; 3, CPCC 206391^T; 4, CPCC 206435^T; 5, *K. aerolata* JCM 16377^T; 6, *K. flava* KCTC 19810^T; 7, *K. locipacati* JCM 17313^T; 8, *K. subterranea* KCTC 19937^T; 9, *K. sinensis* KCTC 19790^T. TR, Trace(<0.5 %); -, not detected; ND, no data. Summed Feature 3* contains $C_{16:1}$ $\omega 7c$ and/or $C_{16:1}$ $\omega 6c$. Summed Feature 6* contains $C_{19:1}$ $\omega 9c$ and/or $C_{19:1}$ $\omega 11c$. Summed Feature 9* contains iso-$C_{17:1}$ $\omega 9c$ and/or $C_{16:0}$ 10-methyl. The data for strains *K. locipacati* DMZ1^T and *K. aerolata* 5317S-21^T were obtained from the the Weon et al.[13] and Shin et al.[10]. All other data were taken from this study.

Supplementary table S5. Biosynthetic gene clusters predicted by AntiSMASH in Strains strains CPCC 206391^{T}, CPCC 206435^{T}, CPCC 206453^{T} and CPCC 206450^{T}.

Strain	Region	Type	From	To	Most similar known	cluster	Similarity
CPCC 206391^{T}	Region 2.2	NAPAA	581,513	615,412	ε-Poly-L-lysine	NRP	100%
	Region 2.4	T3PKS	1,030,055	1,071,158	alkylresorcinol	Polyketide	100%
CPCC 206435^{T}	Region 7.1	T3PKS	19,960	61,042	alkylresorcinol	Polyketide	100%
CPCC 206450^{T}	Region 1.1	T3PKS	1,287,895	1,329,010	alkylresorcinol	Polyketide	100%
	Region 1.2	T3PKS	884,974	926,044	alkylresorcinol	Polyketide	100%
CPCC 206453^{T}	Region 2.3	NI-siderophore	475,204	504,982	desferrioxamine E	Other	100%
	Region 7.1	NAPAA	23,487	57,341	ε-Poly-L-lysine	NRP	100%
